# Taking stock: Is gene drive research delivering on its principles?

**DOI:** 10.12688/gatesopenres.15323.1

**Published:** 2024-02-22

**Authors:** Aaron J. Roberts, Kristy Hackett, Isabelle Coche, Stephanie L. James, Katherine Littler, Michael Santos, Claudia I. Emerson

**Affiliations:** 1 Institute on Ethics & Policy for Innovation, McMaster University, Hamilton, ON, L8S 4L8, Canada; 2 Philosophy, McMaster University, Hamilton, ON, L8S 4L8, Canada; 3 Emerging ag inc., Dugald, Manitoba, R0E 0K0, Canada; 4 GeneConvene Global Collaborative, Foundation for the National Institutes of Health, Bethesda, Maryland, 20852, USA; 5 Global Health Ethics & Governance Unit, Research for Health, World Health Organization, Geneva, Switzerland; 6 Medicine, McMaster University, Hamilton, ON, L8S 4L8, Canada

**Keywords:** Gene Drive, Ethics, Governance, Capacity Building, Biotechnology, Synthetic Biology, CRISPR

## Abstract

Gene drive technology has been recognized for its potential to provide durable and cost-effective solutions for previously intractable problems in public health, conservation, and agriculture. In recognition of the rapid advances in this field, in 2016 the U.S. National Academies of Sciences, Engineering, and Medicine issued a report making several recommendations aimed at researchers, funders, and policymakers for the safe and responsible research and development of gene drive technology. Subsequently, in 2017 sixteen global organizations self-identifying as sponsors and supporters of gene drive research became public signatories committed to the ‘Principles for Gene Drive Research’ which were inspired by the report’s recommendations.

Herein we reflect on the progress of gene drive research in relation to the ethical principles laid out and committed to by the signatories to the Principles. Our analysis indicates high levels of alignment with the Principles in the field of gene drive research. The manuscript also discusses the Gene Drive Research Forum, which had its genesis in the publication of the Principles. Discussions between participants at the latest meeting of the Forum point to the work that lies ahead for gene drive research in line with the Principles. Going forward the gene drive research community can productively focus on: i) safety and efficacy criteria for open release, ii) risk assessment frameworks and methods, iii) more downstream technical, regulatory and policy considerations for field evaluations and implementation, iv) continued transparency and developing mechanisms of accountability, and v) strengthening capacity in locales of potential release and expected drive spread.

## Disclaimer

The views expressed in this article are those of the author(s). Publication in Gates Open Research does not imply endorsement by the Gates Foundation.

## Introduction

Gene drive is a process, either naturally occurring or genetically engineered, whereby a particular gene or genetic construct is able to enhance its own inheritance so that it becomes increasingly prevalent in the population over successive generations ^1^. It has been recognized for its potential to provide durable and cost-effective solutions for previously intractable problems in public health, conservation, and agriculture ^2^.

The first demonstrations that synthetic gene drive systems can function for population modification or suppression of malaria-transmitting mosquitoes were published several years ago ^3, 4^. Shortly thereafter, a National Academies of Science, Engineering, and Medicine (NASEM) report called for ethics and governance work to fill critical gaps in the gene drive research policy space ^2^. “Principles for gene drive research” ^5^ (hereafter, ‘the Principles’) were published in 2017, and were developed in response to NASEM recommendations aimed specifically at funders of gene drive research and development. Recognizing their responsibility in shaping the field, sponsors and supporters of gene drive technology came together to develop the Principles to lay the groundwork for practical, ethical, and responsible scientific advancement in the field ( Box 1).

**Box 1. Principles for gene drive research.**

1. Advance quality science to promote the public good

2. Promote stewardship, safety, and good governance

3. Demonstrate transparency and accountability

4. Engage thoughtfully with affected communities, stakeholders, and publics

5. Foster opportunities to strengthen capacity and education

Thirteen global organizations self-identifying as sponsors and supporters of gene drive research committed to the Principles as founding public signatories; three additional organizations became signatories of the Principles post-publication. This demonstrated explicit commitment of resources to align with and implement the Principles, creating incentive structures for ‘first-movers’ to forge responsible pathways towards development of gene drive technology, and helped to establish a community of practice anchored in high scientific and ethical standards. Concurrent with publication of the Principles, the *Gene Drive Research Forum* (hereafter, ‘the Forum’) formally launched with the goal of providing opportunities for the gene drive research community to share knowledge, and “respectfully and openly consider, discuss, and debate important, challenging, controversial, or overlooked gene drive technology-related issues” ^6^. Since then, members of the Forum have worked together to implement the Principles in their work and meet annually to discuss progress and ongoing challenges.

 Six years onward, most advances in gene drive reflect technical developments in the lab, particularly on gene drive modified mosquitoes for use in malaria elimination, and notable developments in gene drive modified rodents for conservation. Multiple mosquito projects are nearing technical preparedness for field evaluations, bringing pressing policy questions to the forefront. At the 2022 annual meeting of the Forum, the gene drive research community reflected on progress made in the field, paying special attention to whether and how the Principles have been implemented, and the work that remains to harness the potential of gene drive technology to address challenges across public health, conservation, and agriculture. Herein we report on the results of that exercise, and ‘take stock’ of where we are, and where we are going, in gene drive research.

## Annual *Gene Drive Research Forum*: Principles at Work

The aim of the Principles was to mobilize a community of practice around a set of explicitly agreed upon norms that articulated a baseline of general expectations within the gene drive research community. They were crafted to offer broad guidance (“soft policy”) in the interest of avoiding poor decision-making that could result in ‘missed steps’ and/or ‘missteps’ in the further development of this technology. Launch of the Forum coincided with (was in part motivated by) publication of the Principles with a focus on funders of gene drive research, and since 2018 it has grown to also include a large contingent of researchers. Building on the momentum of the Principles, research groups have committed to similar principles ^7– 9^ and work on ethics and governance has ramped up in alignment with the Principles ^10– 14^. The Forum encourages and enables transparency and consensus building and facilitates the establishment of community norms. The annual meeting brings together major stakeholders in the gene drive research community: funders, developers, policymakers, end-users, to discuss the most pressing contemporary issues across the fields of public health, conservation, and agriculture. This is critical because governance pathways must be developed in tandem with the technology itself. Topics discussed at each meeting and summaries of the proceedings are publicly available ^6^. The GeneConvene Global Collaborative, based at the US Foundation for the National Institutes of Health (FNIH), hosts the Forum and makes information widely and freely available to the community and broader public; knowledge sharing and exchange is further facilitated through webinars, workshops and other fora ^6^. It represents a continuous instantiation of the principled commitments to advance quality science, to promote the public good, be transparent, to engage thoughtfully, and to strengthen capacity and education.

## Looking back: How far have we come?

Several gene drive developments since 2017 explicitly align with the goals and spirit of the Principles. For instance, many scientific developments reflect the principle that quality science be advanced in service of the public good (Principle 1). To date, most advanced gene drive research programs focus on applications for public health, particularly on reducing the transmission of mosquito-borne diseases. These include Target Malaria ^15^, University of California Malaria Initiative (UCMI) ^16^, and Transmission Zero ^17^ with public health missions, and the Genetic Biocontrol of Invasive Rodents (GBIRd) program for conservation ^18^. Researchers are developing various mechanisms to address safety and control concerns ^19– 21^, and build knowledge and practice in stakeholder engagement to support responsible research ^7, 8, 10, 22, 23^. Recent development of a world-first proof of concept for the control of invasive mice using t-CRISPR signals continuing promising scientific advances in gene drive research for environmental conservation ^24^.

The second Principle advises to promote stewardship, safety, and good governance. There have been several published works and coordination efforts advancing these goals over the last six years, and development along these lines continues ^7, 11– 13, 25^. We have witnessed increased considerations of the requirements for robust risk assessment for gene drive ^11, 26– 29^ in tandem with a growing recognition of how existing governance and risk assessment mechanisms can be utilized for assessment of gene drive modified organisms (GDMO) ^27, 30, 31^. There is a general understanding amongst Forum members that additional aspects of gene drive governance must be clarified as we draw closer to initial field evaluations.

Organizations funding gene drive research have established policies for data sharing. Whether additional standards of transparency and accountability for gene drive research are required (Principle 3) remains a matter of ongoing discussion. However, the field’s track record in this regard is commendable thus far, as demonstrated by the number of technical and risk assessment reports made publicly available in scientific journals, conferences, and online (available through https://www.geneconvenevi.org/ and project websites). After publication of the Principles, attention focussed immediately on instantiating this Principle. A Forum working group was formed to study the need for a specific registry of gene drive projects, and this theme anchored discussions of the Forum’s 2018 annual meeting. Ideas surfaced around registries, and mechanisms of communication. These discussions continued over the last five years and led to another thematic exploration at the Forum’s 2019 annual meeting, as well as a workshop and publication exploring the pros and cons ^32^.

Thoughtful engagement with affected communities, stakeholders, and publics constitutes the fourth Principle, and perhaps no other Principle has received more effort and attention. The last six years have seen a remarkable number of publications highlighting new frameworks, approaches, and best practices in engagement for gene drive research. Notably, two major research consortia, Target Malaria and UCMI, have published lessons from their extensive community engagement efforts ^8, 10, 22, 23^. Projects exploring the potential of gene drive technologies for conservation of biodiversity also are actively pursuing stakeholder engagement activities ^33^. This scholarship has informed theory and practice of community engagement, both within the gene drive community and beyond. We have also seen the instantiation of two significant engagement initiatives arise, the GeneConvene Global Collaborative ^34^ and the Outreach Network for Gene Drive Research ^35^, which have hosted several freely available public webinar series with experts as well as engagement meetings with key stakeholders, including representatives of Indigenous communities, and conservation thought leaders. The extensive and robust engagement activities of various gene drive research projects contribute broadly to the goals of all the Principles.

Lastly, we have witnessed meaningful capacity strengthening and education with respect to preparedness for responsible regulation and management of GDMO (Principle 5). This includes regulatory capacity-building such as workshops on problem formulation for risk assessment supported by The African Union Development Agency (AUDA-NEPAD) and FNIH ^26^. AUDA-NEPAD and the West Africa Health Organization (WAHO) established the West Africa Integrated Vector Management Programme (WA-IVM) “to promote a multi-sectoral approach in building robust regulatory systems for genetically based vector control applications” ^25^. The Pan African Mosquito Control Association (PAMCA) is building capacity and providing educational opportunities around gene drive research through their African Gene Drive for Vector Control (AGDVC) network and associated events ^36^. The African Genetic Biocontrol Consortium sponsors opportunities for in-depth discussions and training to promote Africa’s self-determination in decision-making about genetic biocontrol approaches such as gene drive ^37^. In partnership with the University of São Tomé and Príncipe (USTP) and the Ministry of Health, UCMI developed a new molecular biology research laboratory located on the USTP campus ^38^. Through collaboration with Target Malaria, new Arthropod Containment Level-2 (ACL-2) labs equipped to work on genetically modified insects were built in Uganda ^39^ and Burkina Faso ^40^. Also in collaboration with Target Malaria, the University of Ghana has a new insectary ^41^ and the Universite des Sciences, Technologies et Techniques (USTTB) in Mali is establishing an African Centre for Excellence in Molecular Engineering to build on and provide training in cutting-edge molecular and genetic engineering to other African researchers ^42^. Earlier this year Transmission Zero generated the first transgenic mosquito created in Africa at Tanzania’s Ifakara Health Institute ^43^. In addition to regulatory and scientific capacity building, we have also seen increased attention to ethics. Many scientific panels include ethics considerations, and guidance documents explicitly focus on exploring important ethical issues related not only to the conduct of gene drive research, but also importantly, the consideration of broader environmental, social, and economic impacts of gene drive and its applications ^11, 25^. 

## Looking forward: what work is left to do to fulfil the vision of the principles?

The last six years have seen much progress. Yet, many countries with difficult and pressing problems which stand to benefit from gene drive technology, whether with a high burden of vector borne disease or species on the brink of extinction due to invasive alien species, must weigh the risks and potential benefits of its use. For many, circumstances remain that would benefit from additional preparation for decision-making about the introduction of GDMO. Much more must be done to expand leadership and capacity in science, ethics, regulation, and oversight within these contexts.

The Principles agreed to in 2017 remain relevant today. What remains to be done to align ongoing gene drive research with them? For example, as gene drive research and development proceeds and the applications under investigation broaden, has a real need been identified for additional gene drive-specific governance mechanisms? Is ongoing work needed to satisfy the transparency and accountability Principles? For instance, there has been much discussion, but no clear resolution, on the question of whether regional or international oversight might be appropriate for certain gene drive applications ^44– 48^. On the matter of accountability, contextually appropriate plans for post-release monitoring, as well as understandings of liability and agreements regarding potential redress need fleshing out and elaboration. The African Union and others have called attention to a need for more work to be done to strengthen capacity and education efforts for gene drive technology ^49^. Planning and predicting the scope (i.e. target and expected) of GDMO geographic spread will be different for individual gene drive products according to their characteristics and target contexts, but has arisen as an area of potential tension between the tendencies of environmental regulation to focus on risk and exposure mitigation, and (e.g., in the case of gene drive modified mosquitoes for malaria elimination) the public health motivated desire for cost-effectiveness and durability through spread and persistence. How can we reconcile this tension?

While no GDMO have been released to date, some research groups are quickly approaching technical readiness for field evaluations, only making these questions more pressing. Box 2 provides a non-exhaustive list of work the Forum identified as worthy of prioritization in furthering responsible research for gene drive technology.

**Box 2. Gene drive research forum areas for further investigation.**

• Mapping the existing relevant governance and regulatory landscape – including clarifying requirements for post-release monitoring, liability, and redress – and identifying any relevant gaps.

• Bettering our understanding of how modelling can be used to predict safety and efficacy outcomes, including how best to evaluate uncertainty.

• Developing guidance to support research projects in determining appropriate scope and nature of engagement for field evaluations of GDMO – e.g. whom, and within what geographic range, to engage, and how?

• Understanding the safety and efficacy characteristics that would be necessary to warrant field release of GDMO in the context of research.

• Understanding other ‘readiness’ requirements for field release: technically, politically (e.g. regulations), and socially/culturally.

• Considering and reconciling potential challenges in operational research, which could be different for self-sustaining and self-limiting technologies (e.g. different degrees of incrementality of field evaluations available).

• Consolidating learnings and approaches developed by different projects and identifying opportunities to apply them more efficiently and consistently.

• Developing guidance regarding commercialization of gene drive applications.

## Conclusion

The field has moved rapidly on several fronts over the last six years, particularly on technical developments and the science of engagement. The Principles have been invaluable for shaping and setting high scientific and ethical standards. They remain more relevant than ever as in the next five years we anticipate cautious progression out of the lab and into contained/controlled field evaluations of gene drive modified mosquitos for malaria elimination.

Our analysis shows that substantive efforts have been made to instantiate *all* of the Principles within the gene drive research community. Noteworthy progress has been made with respect to scientific advancement for the public good (P1), policy developments to promote stewardship, safety and good governance (P2), and considerable discussion and enactment of appropriate transparency and accountability practices (P3). Efforts to engage affected communities, stakeholders and publics have been substantial and are ongoing (P4), and a variety of initiatives aimed at building and strengthening capacity in science, ethics, biosafety, and regulation (P5) have taken root over the last six years. But much more needs to be done.

Going forward the gene drive research community can productively focus on: i) safety and efficacy criteria for open release, ii) risk assessment frameworks and methods, iii) more downstream technical, regulatory and policy considerations for field evaluations and implementation, iv) continued transparency and developing mechanisms of accountability, and v) strengthening capacity in locales of potential release and expected drive spread. These priorities will be critical to support readiness to take the next steps in GDMO development.

## Abbreviations

ACL-2:                 Arthropod Containment Level-2

AGDVC:              African Gene Drive for Vector Control

AUDA-NEPAD:  African Union Development Agency

CRISPR:              Clustered Regularly Interspaced Short Palindromic Repeats

FNIH:                  Foundation for the National Institutes of Health

GBIRd:               Genetic Biocontrol of Invasive Rodents

GDMO:              Gene drive modified organism

NASEM:            U.S. National Academies of Sciences, Engineering, and Medicine

NIH:                   U.S. National Institutes of Health

PAMCA:             Pan African Mosquito Control Association

UCMI:                University of California Malaria Initiative

USTP:                 University of São Tomé and Príncipe

USTTB:              Universite des Sciences, Technologies et Techniques

WAHO:               West Africa Health Organization

WA-IVM:            West Africa Integrated Vector Management Programme

## Data Availability

No data are associated with this article.
