# Supplementary material for: Taking stock: Is gene drive research delivering on its principles?
Source: Gates Open Res. 2024 Feb 22;8:14. [Version 1] doi: 10.12688/gatesopenres.15323.1 (PMC11259591; doi:10.12688/gatesopenres.15323.1)
Supplement: Peer review history

# Reviewer report: Reviewer response for version 1

**DOI:** 10.21956/gatesopenres.16671.r36082

**Published:** 2024-07-22

**Authors:** Alexander Franz

**Affiliations:** 1 University of Missouri, Columbia, Missouri, USA

n 2016-2017, a group of authors defined the 5 Principles (of Gene Drive Research: 1. quality science; 2. stewardship, safety, & governance; 3. transparency & accountability; 4. community engagement; 5. capacity building & education) as a response to an earlier U.S. National Academies of Sciences, Engineering, and Medicine (NASEM) recommendation.

 Now, 7-8 years later, a similar group of authors is presenting a follow-up paper in which they look at the progress of gene drive research achieved during the timespan to assess to what extend the 5 Principles have been fulfilled or taken into consideration by researchers, funding organizations, policy makers, and regulatory bodies.

 The authors declare that relatively high levels of fulfillment/consideration have been accomplished for Principles 3-5 and to a somewhat lesser extent for Principles 4 & 5. Finally, the authors suggest what all needs to be done to accomplish higher levels of fulfillment of the Principles in the near future, especially in view of P 4 & 5.

 The manuscript is well written and clearly structured.   

 Apart from that, the paper lacks concrete examples of gene drive (GD) design principles, population control strategies, and GD field release scenarios. Such examples could be helpful to address the risk/cost-benefit question.  

 Regarding Principles 1 &2, researchers should conduct gene drive experiments in safe containment facilities to avoid accidental release of modified GD bearing individuals (safe practice). Furthermore, researchers should also think of transgene designs or strategies that will allow for the potential recall of a particular GD following its field release if unexpected complications regarding the GD or its linked effector arise.

 Regarding P 3, it may be necessary to have an international body (WHO, or the like) to come up with a regulatory/legal framework regarding GD research and potential release scenarios. For example: Following their release, GD bearing mosquitoes may get unintentionally transported/dispersed into “non-target” areas along with human trade & traffic or due to weather incidents, perhaps even across continents. From a legal point-of-view, the situation could become problematic, if those manipulated mosquitoes would accidentally show up in “non-target” regions and start biting the unaware/unprepared human population.      

 Regarding P5, the GD recipient countries/communities should be sufficiently trained and educated so that they will be able to manage the GD field implementation, its safe-guarding, surveillance over time, and quality assessment by themselves, meaning that they eventually become independent from the technology transfers of the original GD developers.

Does the article adequately reference differing views and opinions?

Yes

Are all factual statements correct, and are statements and arguments made adequately supported by citations?

Yes

Is the Open Letter written in accessible language?

Yes

Where applicable, are recommendations and next steps explained clearly for others to follow?

Yes

Is the rationale for the Open Letter provided in sufficient detail?

Yes

Reviewer Expertise:

vector biology, arbovirology

I confirm that I have read this submission and believe that I have an appropriate level of expertise to confirm that it is of an acceptable scientific standard.

# Reviewer report: Reviewer response for version 1

**DOI:** 10.21956/gatesopenres.16671.r36085

**Published:** 2024-07-22

**Authors:** Georg Oberhofer

**Affiliations:** 1 California Institute of Technology, Pasadena, California, USA

This manuscript examines the advancement of gene drive science in relation to the five principles for responsible research established by the National Academies of Sciences, Engineering, and Medicine (NASEM) in 2017. The authors highlight progress made towards each principle and identify areas requiring further investigation.

 Comments:

 "Researchers are developing various mechanisms to address safety and control concerns19–21"

 The authors cite a paper on how to reverse/stop a drive, a review, and a theoretical modeling paper. What about high threshold and self-limiting (split) drives? Both of these concepts have already been implemented in the lab. Self-limiting drive technologies are briefly mentioned at the end of the paper in the outlook box. Would also move this to the next paragraph discussing principle 2 (safety).

 "There is a general understanding amongst Forum members that additional aspects of gene drive governance must be clarified as we draw closer to initial field evaluations."

 What specific aspects need further clarification?

 "Whether additional standards of transparency and accountability for gene drive research are required (Principle 3) remains a matter of ongoing discussion."

 The community would benefit from knowing what additional standards are being discussed that haven't been implemented yet.

 The authors conclude that progress was made towards all 5 principles. Did any areas see slower progress, demanding a greater focus/effort moving forward?

Does the article adequately reference differing views and opinions?

Yes

Are all factual statements correct, and are statements and arguments made adequately supported by citations?

Yes

Is the Open Letter written in accessible language?

Yes

Where applicable, are recommendations and next steps explained clearly for others to follow?

Yes

Is the rationale for the Open Letter provided in sufficient detail?

Yes

Reviewer Expertise:

Genetics, Gene drive

I confirm that I have read this submission and believe that I have an appropriate level of expertise to confirm that it is of an acceptable scientific standard, however I have significant reservations, as outlined above.

# Reviewer report: Reviewer response for version 1

**DOI:** 10.21956/gatesopenres.16671.r36087

**Published:** 2024-07-19

**Authors:** Philip T Leftwich

**Affiliations:** 1 University of East Anglia, Norwich, England, UK

In this letter, the authors critically appraise and reflect on the recent progress of gene drive research and emphasis the value of open discussion. This discussion is crucial in progressing gene drive technologies and ensuring governance in line with ethical principles, especially as these technologies are getting closer to field readiness. This letter makes a valuable and substantive contribution to managing gene drive research. Still, I feel that in many areas, more information is needed to provide a comprehensive understanding of progress. 

1. In the introduction, the authors provide a gene drive definition that is in line with the cited source but neglect to mention that this definition correctly covers gene drive as a process but can equally validly be defined as an 'object' or 'intention'. 
2. The information provided on the Annual Gene Drive Research Forum lacked detail. It would be helpful to evaluate the relative successes of this forum.
3. The authors discuss and identify the current sponsors and supporters of gene drive research who have committed to the Principles. However, evaluating these organizations' global distribution and impactful scope is of considerable interest. Are more organizations needed to sign up to these Principles in order for it to be a globally applied standard? Is this an aim or target for the Gene Drive Research Forum, and how realistic is it to have a global set of conventions or standards?
4. There is limited discussion on safety and control concerns; three references are given, with a weighting towards gene drive reversal rather than focusing on containment or limitation by design. 
5. The t-CRISPR development is of interest but without context or explanation as to why this particular development signals a promising scientific advance.
6. When discussing the second Principle, the additional governances requiring discussion are not given.
7. When discussing gene-drive research funding, no information is provided on the established policies for data sharing or the expected requirements for data gathering standards. Without this it is difficult to evaluate the scope of current standards. 
8. I find reference to the number of technical and risk assessment reports as an indicator of standards to be a low-quality metric.
9. When discussing engagement with communities and stakeholders, I found it surprising to see no mention of the World Mosquito Program. This is a missed opportunity in my opinion to compare strategies and successes. 
10. Looking forward - when wrapping up, there is little roadmap regarding how expanded leadership, capacity and regulation might be established or expanded. 

I recognize that the authors must cover vast literature and rapidly evolving developments within a short letter. However, the lack of detail in many areas made it difficult to effectively reflect on milestones and progress.

Does the article adequately reference differing views and opinions?

Partly

Are all factual statements correct, and are statements and arguments made adequately supported by citations?

Partly

Is the Open Letter written in accessible language?

Partly

Where applicable, are recommendations and next steps explained clearly for others to follow?

Partly

Is the rationale for the Open Letter provided in sufficient detail?

Yes

Reviewer Expertise:

Insect genetics, transgenics, behavioural ecology

I confirm that I have read this submission and believe that I have an appropriate level of expertise to confirm that it is of an acceptable scientific standard, however I have significant reservations, as outlined above.

# Reviewer report: Reviewer response for version 1

**DOI:** 10.21956/gatesopenres.16671.r36086

**Published:** 2024-04-16

**Authors:** Roberto Galizi

**Affiliations:** 1 Keele University, Keele, England, UK

The open letter from Roberts et al provides a critical appraisal and reflection on recent progress of gene drive research. The authors summarise the related ethical principles and appraise alignment to these of current/recent research, following establishment of proof of concept in the laboratory. Open discussion has and continues to warrant progress and governance in line with the principles and delineate focal priorities while gene drive technologies are getting closer to field readiness. 

 I have enjoined reading the manuscript and only have a few minor comments that the authors may want to consider addressing if and where feasible.

1. The authors write “There is a general understanding amongst Forum members that additional aspects of gene drive governance must be clarified as we draw closer to initial field evaluations.”. However there is no specific reference to what these might be or specifically examined in the manuscript (could be Box 2?).
2. Related to the above, the letter refers and rely consistently on the Gene Drive Research Forum and Gene Convene webpages, also as source of references. I am wondering if these hyperlinks may remain active and/or web content will continue to remain up to date in the mid/long term or, alternatively, there could be other sources that could be used as reference/s here or for other web content cited.
3. The authors write “Organizations funding gene drive research have established policies for data sharing.” Might be useful to cite and/or quote some examples if feasible, this seem particularly relevant to the open discussion.   
4. The authors write “…the public health motivated desire for cost-effectiveness and durability through spread and persistence. How can we reconcile this tension While no GDMO have been released to date, some research groups are quickly approaching technical readiness for field evaluations, only making these questions more pressing”. Something that might not be fully clear or worth clarifying is the complexity (if not impracticability in many cases) to perform “field evaluation” for the most cost-effective (highly invasive) gene drives. This is somehow captured in Box 2 – point 5 “Considering and reconciling potential challenges in operational research, which could be different for self-sustaining and self-limiting technologies (e.g. different degrees of incrementality of field evaluations available)” although not particularly clear in the main text.
5. Some of the other points in Box 5, e.g. “commercialization of gene drive applications” and “‘readiness’ requirements” could be further explained and/or examined in the main text.

Does the article adequately reference differing views and opinions?

Yes

Are all factual statements correct, and are statements and arguments made adequately supported by citations?

Yes

Is the Open Letter written in accessible language?

Yes

Where applicable, are recommendations and next steps explained clearly for others to follow?

Yes

Is the rationale for the Open Letter provided in sufficient detail?

Yes

Reviewer Expertise:

Infectious diseases, insect genetics, gene drives

I confirm that I have read this submission and believe that I have an appropriate level of expertise to confirm that it is of an acceptable scientific standard.

# Reviewer report: Reviewer response for version 1

**DOI:** 10.21956/gatesopenres.16671.r36080

**Published:** 2024-04-11

**Authors:** Simon W Baxter

**Affiliations:** 1 The University of Melbourne, Melbourne, Victoria, Australia

Here the authors return to a theme previously summarised in their 2017 article “Principles for gene drive research” (ref 5) and present evidence for the progress that has been made in the past 6 or 7 years. The manuscript clearly outlines the five Principles for gene drive research, which draws attention to these pillars attempting to uphold accountability, transparency and many other aspects of this field. The authors then discuss whether gene drive research is delivering on the Principles, as stated in the title.

 This article is written in a style that will be accessible to a broad audience and is likely to be a useful point of reference for readers wanting information regarding regulation of synthetic gene drives. While this is a useful summary, I did find the language was often quite vague, making it difficult for the reader to understand any specific evidence being highlighted. Phrases like “several years ago”, “notable developments”,  “a large contingent of researches”, “continuing promising scientific advances”, “stakeholder engagement activities” are all fairly vague. For example 16 countries have signed up to the principles but we don’t know who they are.

 In paragraph 2, the authors refer to the analysis they have undertaken, which shows “high level of alignment with the Principles in the field of gene drive research”. It seems like the analysis was a literature review, although there isn’t a summary of this data which would increase opportunities to cite this manuscript.

 The first principle (page 4), advancing quality science to promote the public good, highlights gene drives in mice and Anopheles as two examples. Perhaps scientific detail explaining some of the safety mechanisms in is beyond the overview this review provides, but I think it is important to highlight that CRISPR technology has been quite challenging to achieve in many species including mice and other disease vectors such as *Aedes* mosquitoes. There are many technical challenges and this should be highlighted.

 The second principle (page 4), promoting stewardship, safety and good governance, doesn’t provide the reader with any clear information. It would be helpful to explain what the progress has been made since 2017 that are outlined in the references. If there are “additional aspects of gene drive governance” that require clarification, perhaps you could provide some of the key areas.

 Very little detail is provided for principle 3, aside from a website link and reference 32. Perhaps it would be worth highlighting what the “pros and cons” were discussed in the various workshops and meetings regarding transparency, accountability and communication for gene drive research. What are the strengths and weakness of a gene drive registry?

 Discussion regarding Principle 4, engaging with stakeholders and publics, might benefit from explaining specific strategies for engaging with (indigenous) stakeholders. Principle 5 addressed capacity for gene drive applications in Africa, and to some extent strengthening of gene drive education. Is it necessary to include acronyms of all the institutes and organisations?

 Box 2 provides some very interesting focal points for further debate and the article could be a useful reference to support justification of Forum meetings in years to come.

Does the article adequately reference differing views and opinions?

Partly

Are all factual statements correct, and are statements and arguments made adequately supported by citations?

Yes

Is the Open Letter written in accessible language?

Yes

Where applicable, are recommendations and next steps explained clearly for others to follow?

Partly

Is the rationale for the Open Letter provided in sufficient detail?

Yes

Reviewer Expertise:

Genetic and genomic analysis of insect pests

I confirm that I have read this submission and believe that I have an appropriate level of expertise to confirm that it is of an acceptable scientific standard, however I have significant reservations, as outlined above.
